# Analysis of Slow (Theta) Oscillations as a Potential Temporal Reference Frame for Information Coding in Sensory Cortices

**DOI:** 10.1371/journal.pcbi.1002717

**Published:** 2012-10-11

**Authors:** Christoph Kayser, Robin A. A. Ince, Stefano Panzeri

**Affiliations:** 1 Max Planck Institute for Biological Cybernetics, Tübingen, Germany; 2 Bernstein Center for Computational Neuroscience, Tübingen, Germany; 3 Institute of Neuroscience and Psychology, University of Glasgow, Glasgow, United Kingdom; 4 Center for Neuroscience and Cognitive Systems @UniTn, Istituto Italiano di Tecnologia, Rovereto, Italy; University of Oxford, United Kingdom

## Abstract

While sensory neurons carry behaviorally relevant information in responses that often extend over hundreds of milliseconds, the key units of neural information likely consist of much shorter and temporally precise spike patterns. The mechanisms and temporal reference frames by which sensory networks partition responses into these shorter units of information remain unknown. One hypothesis holds that slow oscillations provide a network-intrinsic reference to temporally partitioned spike trains without exploiting the millisecond-precise alignment of spikes to sensory stimuli. We tested this hypothesis on neural responses recorded in visual and auditory cortices of macaque monkeys in response to natural stimuli. Comparing different schemes for response partitioning revealed that theta band oscillations provide a temporal reference that permits extracting significantly more information than can be obtained from spike counts, and sometimes almost as much information as obtained by partitioning spike trains using precisely stimulus-locked time bins. We further tested the robustness of these partitioning schemes to temporal uncertainty in the decoding process and to noise in the sensory input. This revealed that partitioning using an oscillatory reference provides greater robustness than partitioning using precisely stimulus-locked time bins. Overall, these results provide a computational proof of concept for the hypothesis that slow rhythmic network activity may serve as internal reference frame for information coding in sensory cortices and they foster the notion that slow oscillations serve as key elements for the computations underlying perception.

## Author Summary

Neurons in sensory cortices encode objects in our sensory environment by varying the timing and number of action potentials that they emit. Brain networks that ‘decode’ this information need to partition those spike trains into their individual informative units. Experimenters achieve such partitioning by exploiting their knowledge about the millisecond precise timing of individual spikes relative to externally presented sensory stimuli. The brain, however, does not have access to this information and has to partition and decode spike trains using intrinsically available temporal reference frames. We show that slow (4–8 Hz) oscillatory network activity can provide such an intrinsic temporal reference. Specifically, we analyzed neural responses recorded in primary auditory and visual cortices. This revealed that the oscillatory reference frame performs nearly as well as the precise stimulus-locked reference frame and renders neural encoding robust to sensory noise and temporal uncertainty that naturally occurs during decoding. These findings provide a computational proof-of-concept that slow oscillatory network activity may serve the crucial function as temporal reference frame for sensory coding.

## Introduction

Oscillatory activity generated by local cortical networks is considered to be a crucial component of sensory processing [1], [2], [3] and has been implicated in processes such as the temporal binding of neural assemblies, the control of information flow between areas [4], [5], or the multiplexing of information across different time scales [6], [7]. Theoretical work has also proposed a critical role for slow oscillations in temporally organizing the information carried by prolonged neural responses [8], [9], [10], [11], [12], [13].

Sensory information provided by neural firing patterns about naturalistic stimuli is often stretched over periods of several tens to a few hundreds of milliseconds, and the full information provided by such responses can only be extracted when considering the extended firing pattern as a whole [14], [15], [16], [17]. For example, hippocampal place cells encode the current position of the animal in space, yet meaningful trajectories can only be read out when observing the activity of such populations over periods of several hundreds of milliseconds [18], [19]. In addition, natural stimuli such as sounds or movies entrain cortical activity on slow time scales and require the readout of response patterns over several tens to hundreds of milliseconds to correctly decode different scenes [14], [20], [21], [22]. Such extended and time-varying representations can be correctly interpreted only when the decoder is able to partition the full response into smaller chunks of a few tens of milliseconds, and to correctly position these chunks relative to each other within the neural response and relative to the sensory input [16], [17], [23].

For data analysis, such temporal partitioning is usually performed by aligning single trial responses relative to stimulus onset and dividing them into equally-spaced and precisely stimulus-locked time bins. While this is easily done by the experimenter, it makes the assumption that the decoder has access to a highly precise clock. Aligning neural responses to the stimulus requires the decoder to have precise knowledge about the timing of sensory events (e.g. stimulus onset). In addition, the ability to partition longer spike trains into smaller patterns requires either a nearly perfect representation of time intervals (the analogues of “time bins”) or the ability to represent multiple reference time points during a temporally extended stimulus with high precision. Sensory cortical circuits, however, do not have access to the experimenter's clock and have to rely on intrinsic (either absolute or relative) timing mechanisms [24], [25], [26]. While population responses or the responses of specific subsets of neurons have been suggested as a potential intrinsically available signal of stimulus onset [24], [25], it remains unclear what intrinsic timing signal is exploited to partition longer spike trains. Slow oscillations with cycle lengths of 100 ms or longer (such as delta or theta band rhythms) have been proposed as basis for temporal response partitioning [13], [19]. Oscillation-based partitioning can, for example, be achieved by considering different oscillatory phase angles as partitions of the longer period represented by the full cycle, effectively creating a serial order of partitions within an oscillation cycle. Indeed, work on the hippocampus has suggested that hippocampal theta oscillations can be used as internal temporal reference frame to reconstruct firing assemblies and to decode single neuron's responses [8], [13], [19], . Importantly, such an oscillatory reference frame is intrinsic to the cortical network and its specific timing parameter, i.e. the oscillatory phase, is likely to be directly accessible to the local network [13], [29].

When considering sensory cortical structures, however, the role of oscillations as a network-intrinsic reference has been mostly treated at a conceptual level and the degree to which slow oscillations are useful for partitioning spike trains into temporal units of information remains to be investigated in a quantitative way [10], [13], [30]. Part of this problem is methodological as long spike trains partitioned into a finely timed pattern constitute a high dimensional neural code, for which it is challenging to estimate sensory information due to dimensionality issues and a lack of viable models of sensory encoding [31], [32]. Because of such difficulties, prior work on the complementarity of stimulus information in spikes and the phase of slow oscillations has concentrated mostly on short time scales of a few tens of milliseconds [12], [20], [21]. As a result, previous studies have succeeded in revealing the complementarity of information in instantaneous and stimulus locked spike patterns and oscillatory phase, but did not address whether the phase might provide an effective temporal reference to partition longer responses into informative units over the scale of a few hundreds of milliseconds.

The specific goal of this study is to quantitatively test the hypothesis that slow oscillations can serve as a reference for partitioning spike trains of tens to hundreds of milliseconds into a highly informative code without requiring an external timing reference. Stimulated by the observation that naturalistic stimuli entrain slow cortical activity [6], [33] and that such oscillatory activity likely plays a key role in sensory perception [10], [34] we focus on slow oscillations (<30 Hz) as a putative reference. We systematically compare the information carried by codes that establish temporal relations between spikes either by the binning of spikes into stimulus-locked and equally spaced time bins or by the binning of spikes using the phase of an oscillatory signal. We apply this analysis to evaluate the sensory information carried by single neuron responses recorded in primate auditory and visual cortices during stimulation with long stretches of naturalistic stimuli.

## Results

### Temporal partitioning of neural responses using internal and external reference frames

**Figure 1:**
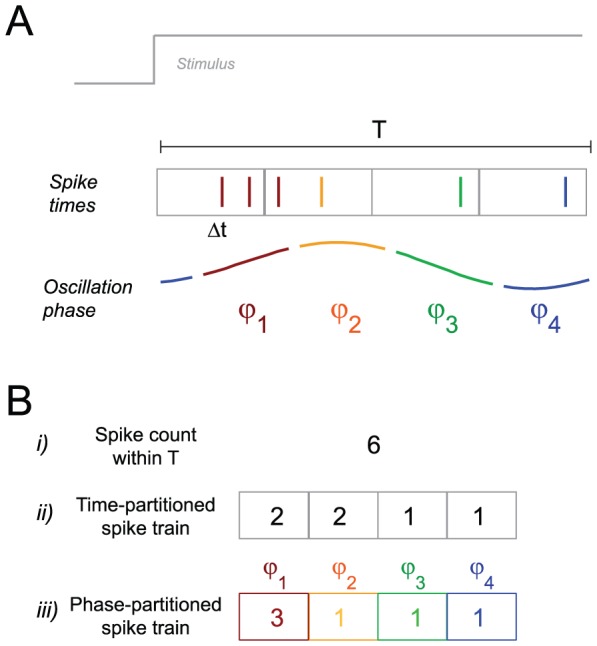
Schematic illustration of temporal partitioning schemes. A) Consider a spike train within a time window T during stimulus presentation. The timing of individual spikes can be measured using a binning procedure relative to stimulus onset (gray boxes). Alternatively, the timing can be measured relative to an intrinsic slow oscillatory signal. Here the phase of such an oscillation was divided into four phase quadrants (φ_i_) and spikes are color-coded by their respective phase angle. B) In this study we consider three codes. i) The spike count, defined as total number of spikes within the window T. ii) A code based on a spike train partitioned using stimulus-locked and equally-spaced time bins (‘time-partitioned’), defined as the vector consisting of the number of spikes per time bin. iii) A code based on the phase-partitioned spike train (‘phase-partitioned’), defined as the vector consisting of the number of spikes per phase range (e.g. phase quadrant, colored).

We begin by illustrating the concept of partitioning a neural response during a time window T using either a stimulus-locked or an oscillatory reference. The timing of individual spikes, such as in the illustration in Fig. 1A, can be measured using a laboratory-based clock, which registers the precise timing of the stimulus (e.g. at t = 0) and of each spike. This stimulus-locked timing is used, for example, when computing a classical peri-event time-histogram (PETH) using a sub-division of the time window T into smaller and equally-spaced time bins of length Δt (Fig. 1A). Counting the number of spikes per bin defines a neural code based on stimulus-locked partitioning. We denote the so defined single-trial response as the time-partitioned spike train (abbreviated as *time-partitioned* in the following Fig. 1B). While this temporal partitioning provides a convenient and frequently used representation of neural responses for analysis, it requires a highly accurate representation of time intervals needed to establish the equally spaced time bins Δt. This information is easily available to the experimenter, but it is not likely to be available to sensory cortical networks.

One way to partition a spike train using a signal intrinsic to the neural network is to use the timing of spikes relative to the phase (the position within an oscillatory cycle) of periodic network activity [18], [20], [21], [35]. Here we consider slow rhythms (e.g. theta range, 2–6 Hz) in local field potentials (LFP) as an oscillatory reference, as oscillations in this frequency range have been implicated in sensory encoding in previous studies [6], [8], [10], [20], [33]. We use the phase of these LFP oscillations (recorded on the same electrode as the spikes) to construct time dependent responses that preserve the sequential order of spikes within the oscillation cycle. In other words, the phase (i.e. the position within a cycle) of the rhythm is used as temporal reference (i.e. as a virtual ‘time axis’) for the temporal organization of responses into distinct but possibly not equally-spaced epochs. In the example we colored four quadrants of the phase angle and labeled spikes falling within each quadrant with the corresponding color (Fig. 1A). The phase-partitioned spike train code (abbreviated as *phase-partitioned*) was constructed, within each time window T, as the number of spikes occurring within each phase quadrant (Fig. 1B). This definition of a neural code is similar to previous studies on hippocampal place cells that have explored the role of theta phase precession in providing information about the animal's location in space [19]. It should be noted that a priori both codes capture distinct and potentially independent aspects of the response. However, if the oscillation is well aligned to the sensory stimulus, both codes will likely carry related patterns of stimulus selective responses. In fact, the main result of our study is that because slow oscillations in auditory and visual cortex are stimulus entrained both codes carry related information and the phase-partitioned code can provide a large proportion of the information that is extracted by time-partitioned responses. However, it does so without relying on a precise and stimulus-locked clock.

For comparison, we also quantified the information provided by the total spike count within the same window. This code provides an estimate of the information that can be extracted without temporally partitioning responses within the time window T and serves as a reference to compare the additional information that can be obtained using the two partitioning schemes above the information available in the spike count. We implemented the spike count using a bin-shuffling procedure that preserves the dimensionality of the time- and phase-partitioned codes and using a 1-dimensional representation. Both yielded very comparable results.

To quantitatively assess the effectiveness of the oscillatory phase in partitioning spike trains in comparison to other codes we used a framework of single trial decoding. We applied this analysis with a wide range of parameters and to different data sets obtained from primary auditory and visual cortices of non-human primates. We compared the stimulus discrimination performance in the phase-partitioned code to the spike count in order to assess the gain by introducing a phase-based temporal partitioning. And we compared the phase-partitioned to the time-partitioned code to judge the performance of the oscillatory phase in partitioning spike trains against an ideal external observer with independent precise knowledge of the time course of both neural and sensory events.

### Comparison of partitioning schemes on auditory cortical data

**Figure 2:**
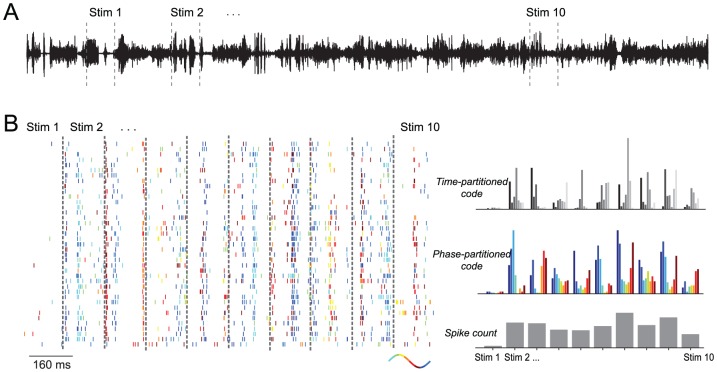
Example data from an auditory cortex neuron illustrating responses in each code. A) Sound wave of the 52 sec acoustic stimulation sequence consisting of various natural and environmental sounds. Dashed lines illustrate the random selection of 10 epochs from the long sound sequence as used for the decoding analysis (epoch duration not to scale). B) Data from one example neuron showing the spike raster (left) and the average response patterns for the three codes (right). The spike raster displays the response to multiple repeats of ten stimulus epochs (concatenated for display purposes). Spikes are color-coded with the phase of the theta (2–6 Hz) band oscillation at the time of spike. The average responses illustrate the trial-averaged responses for each code. Colors indicate the phase bins, gray-scales indicate the time bins.

The auditory system is often faced with a stream of sounds and has to represent individual sound objects within a continuously evolving environment [10], [36]. Examples are individual words in a spoken sentence, a melody in a song or individual sounds appearing in a cacophony of environmental noises. To quantify the performance of each of the proposed codes in such a scenario, we analyzed neural responses recorded from primary auditory cortex of awake animals during the presentation of a 52 second continuous sequence of naturalistic sounds, such as animal calls and environmental sounds (40 responsive neurons recorded from 23 recording sites in three animals, from [20]). To quantify the stimulus discriminability afforded by each code we randomly sampled sets of 10 epochs from the long sound sequence and used these epochs as ‘stimuli’ for the decoding analysis (Fig. 2A). Fig. 2B displays the response of one example neuron to one set of stimulus epochs. The raster plots display the spike trains evoked on individual repeats of the sound sequence. To illustrate the temporal organization of the responses with respect to the oscillatory phase we colored individual spikes according to the phase of the theta LFP (2–6 Hz) at the time of spike. To illustrate the stimulus selectivity of different neural codes, the right-hand panel displays the trial-averaged responses to each stimulus for each code. The stimulus dependence of these average responses is visible in the different profiles of the time or phase-partitioned responses, or the difference in overall spike count across stimuli. We systematically compared the decoding performance across a range of time bins, window durations and frequency bands used to extract the phase, every time averaging decoding performance over 100 sets of randomly selected stimulus epochs to ensure the generality of results across a wide range of acoustic inputs. Across this parameter range, decoding performance was consistently highest using the time-partitioned code and lowest using the total spike count.

**Figure 3:**
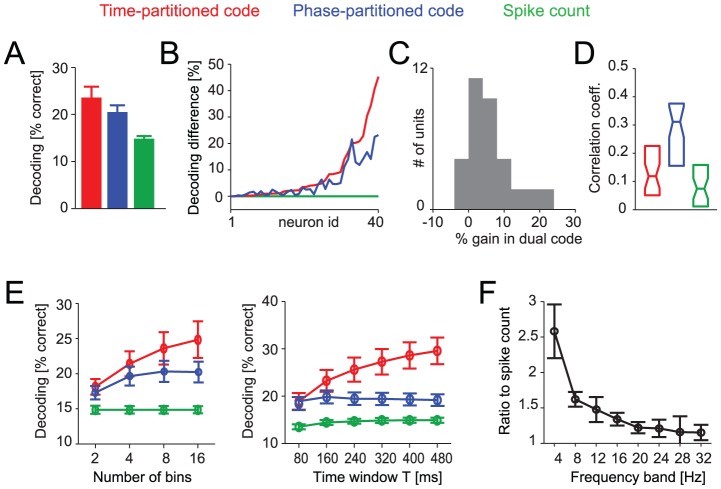
Population results for auditory cortical data. A) Decoding performance across neurons (n = 40, mean and s.e.m.) for each code (N = 8 bins, T = 160 ms window, 2–6 Hz LFP). B) Difference between the decoding performance in time- and phase-partitioned codes and the spike count for each neuron. Neurons are sorted according to the difference for the time-partitioned code (red). Parameters as in A). C) Histogram across neurons of the % gain in decoding performance in the dual time- and phase-partitioned code over the better (for each neuron) of the two individual codes. Parameters as in A). D) Correlation coefficient between the inter-trial phase coherence of the 2–6 Hz LFP and the decoding performance (percentage correct) for individual stimulus epochs used for decoding. Boxplots display the median and quartiles across neurons for each code. Parameters as in A). E) Population averaged decoding performance for different lengths of the stimulus epoch window T (N = 8 bins) and for different numbers of bins (T = 160 ms). F) Ratio of the information in the phase-partitioned code to the information in the spike count when using different frequency bands (4 Hz width) to derive the oscillation phase. Abscissa values indicate center frequencies for each band.

For example, when using 8 bins of a theta band (2–6 Hz) oscillation to divide a 160 ms time window T the decoding performance was 23.6±2% (correct discriminations, chance level 10%) for the time-partitioned code, 20.4±1.5% for the phase-partitioned code and 14.8±0.5% for the spike count (mean ± s.e.m., n = 40 units; Fig. 3A). Differences between all codes were statistically significant (paired t-tests, at least p<10^−3^), showing that temporal response partitioning using the oscillatory phase constitutes a code that affords a higher level of stimulus discrimination than provided by the spike count.

To directly assess the difference in decoding performance between partitioning schemes on a neuron by neuron basis we calculated the relative difference in performance of time- and phase-partitioned codes to the spike count. Given that both time- and phase-partitioned codes implicitly include the spike count, the excess information in either partitioning scheme reflects the amount of stimulus discrimination that is made available by each partitioning scheme above and beyond what is available from the spike count [6], [37]. The result (Fig. 3B) demonstrates a consistent increase of decoding performance when using phase-partitioning over the spike count for each individual unit, and demonstrates that the time-partitioned code provides only a little more information than the phase partitioned code. Indeed, the excess performance in the phase-partitioned codes was close to that of the time-partitioned code (91±2%). Importantly, the excess information in either partitioning scheme over the spike count was highly correlated across neurons (spearman-rank correlation r = 0.87). Good stimulus discrimination afforded by one partitioning scheme hence implies good discrimination performance also in the other.

This correlation of performance across neurons already suggests that both codes effectively capture similar aspects of the neural response. This can be understood intuitively by onsidering the fact that, while the two partitioning schemes rely on the precise alignment of spikes to two potentially very different reference frames (one based on an external timing signal, one based on intrinsic brain activity), in practice the two reference frames are correlated because low frequency oscillations are often entrained by the dynamic environment [20], [33]. This makes it possible that both schemes carry largely similar information. To directly quantify the overlap in decoding performance between the two partitioning schemes we performed additional calculations. First, we calculated the information in a dual-code consisting of both time- and phase-partitioned responses. If these codes would characterize the same or similar response features, the information in the dual-code should exceed the information in the best individual code by only a small amount. However, in case both would characterize independent response aspects, performance in the dual code should by far exceed the best individual code. We found that performance in the dual code was only 4±1% above the best individual code (Fig. 3C), demonstrating a high degree of overlap in stimulus selectivity. Second, we directly investigated the impact of oscillatory trial-by-trial phase alignment on the performance of the phase-partitioned code. Oscillatory phase alignment of the theta LFP was measured for each stimulus epoch using the average phase coherence during that epoch; the phase coherence was computed across trials and averaged over time points during the stimulus epoch. This phase coherence value indicates how well the oscillation is locked relative to the sensory stimulus (hence to the time-partitioning reference) within each epoch. For each unit and for each of the three codes we correlated the phase coherence with the decoding performance across stimulus epochs. This revealed a considerable correlation between phase coherence and decoding performance in the phase-partitioned code (median correlation r = 0.31; Fig. 3D), and (as control) considerably weaker correlations between phase coherence and the performance of the time-partitioned codes (r = 0.1) and the spike count (r = 0.07).

These finding were robust to the number of bins used to divide the time window or the phase range and to the length of the time window T within which the neural response was considered. We varied both parameters independently (Fig. 3E, Supplemental Fig. S1) and found that the phase-partitioned code provided good discrimination performance regardless of whether the time window T was shorter (e.g. 80 ms) or longer (e.g. 320 ms) than a typical cycle of the slow rhythm used to derive the phase (the median cycle length of the 4–8 Hz band was 182 ms across sites).

We also investigated how the information carried by phase-partitioned code depends on the specific frequency band used to derive the phase. Previous work has suggested that stimulus specific information in auditory cortical field potentials is highest for low (<8 Hz) frequency oscillations [20], [33], [38], [39]. We found that this also holds in the present setting, where the oscillatory phase is used to partition longer spike trains into stimulus-specific response patterns. Specifically, we computed the ratio of the decoding performance in the phase-partitioned response relative to the information in the spike count when considering different frequency bands (Fig. 3F). The performance gain in the phase-partitioned code relative to the spike count was largest when deriving the phase from theta-band oscillations (2–6 Hz), mean ratio 2.58±0.38 and was significantly smaller when using e.g. beta (14–18 Hz, ratio 1.34±0.1, t-test p<10^−5^) or higher (26–30 Hz, ratio 1.1±0.08, p<10^−6^) frequency bands. This result was independent of the specific choice of filters used to derive the frequency band (Supplemental Fig. S2A).

### Comparison of partitioning schemes on visual cortical data

**Figure 4:**
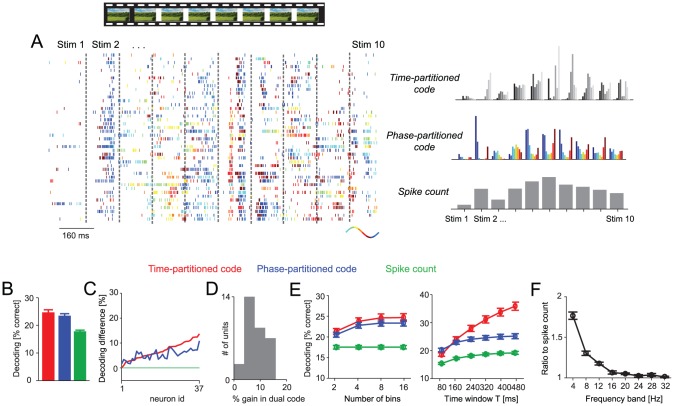
Example and population data for visual cortex data. A) Data from an example neuron showing the spike raster (left) and the average responses for the three codes (right). The spike raster displays the responses to multiple repeats of the video stimulus during ten selected epochs (concatenated for display purposes). Spikes are color coded with the phase of the theta (2–6 Hz) band LFP at the time of spike. The average responses illustrate the trial-averaged responses for each code. Colors indicate the phase bins, gray-scales indicate the time bins. B) Decoding performance across neurons (n = 37, mean and s.e.m.) for the three codes (N = 8 bins, T = 160 ms window, 2–6 Hz LFP). C) Difference between the decoding performance in time- and phase-partitioned codes and the spike count for each neuron. Neurons are sorted according to the difference for the time-partitioned code (red). Parameters as in B). D) Histogram across neurons of the % gain in decoding performance in the dual time- and phase-partitioned code over the better (for each neuron) of the two individual codes. Parameters as in B). E) Population averaged decoding performance for different lengths of the stimulus epoch window T (N = 8 bins) and for different numbers of bins (T = 160 ms). F) Ratio of the information in the phase-partitioned code to the information in the spike count when using different frequency bands (4 Hz width) to derive the oscillation phase. Abscissa values indicate center frequencies for each band.

To test the validity of these findings in a different sensory modality, we repeated the above analysis on data recorded in primary visual cortex during the presentation of commercial color movies (37 responsive neurons recorded from 37 recording sites in four animals). The example data in Fig. 4A illustrate the selectivity of each code for a set of scene epochs extracted from the long (several minutes) video presentation. As for the auditory data, we found that partitioning spike trains using theta range (2–6 Hz) oscillation phase provided significantly better decoding performance than obtained from the spike count (T = 160 ms, 8 bins; spike count: 17.9±0.4%, phase-partitioned code: 23.6±0.7%; paired t-test p<10^−8^; n = 37 units; Fig. 4B). The difference in decoding performance between partitioning based on phase and stimulus-locked time-bins was quantitatively small, though significant (time-partitioned code: 24.8±0.9%; p<0.05). Still, the excess information in the phase-partitioned code over the spike count was nearly that of the excess information in the time-partitioned code (96±2%). Hence, in this dataset partitioning by the oscillation phase provides a code that is nearly as informative as partitioning using stimulus-locked time bins.

As for the auditory dataset we found that performance in both partitioning schemes was highly correlated across neurons (Fig. 4C, median r = 0.83), and that combining both codes provided only 6±0.1% higher performance than the best performing individual partitioning scheme (Fig. 4D). Also as for the auditory dataset, the decoding performance using phase-partitioning was correlated with the oscillatory phase-coherence during the stimulus epoch (median r = 0.26) and similar results were found when using fewer or more bins and when considering time windows of different duration (Fig. 4E). The performance gain in the phase-partitioning relative to the spike count was largest when deriving the phase from low frequency oscillations in the theta frequency range (Fig. 4F). This suggests theta-range rhythms as privileged candidates for intrinsically-available reference frames in sensory cortex.

### Stimulus encoding in face of temporal uncertainty

**Figure 5:**
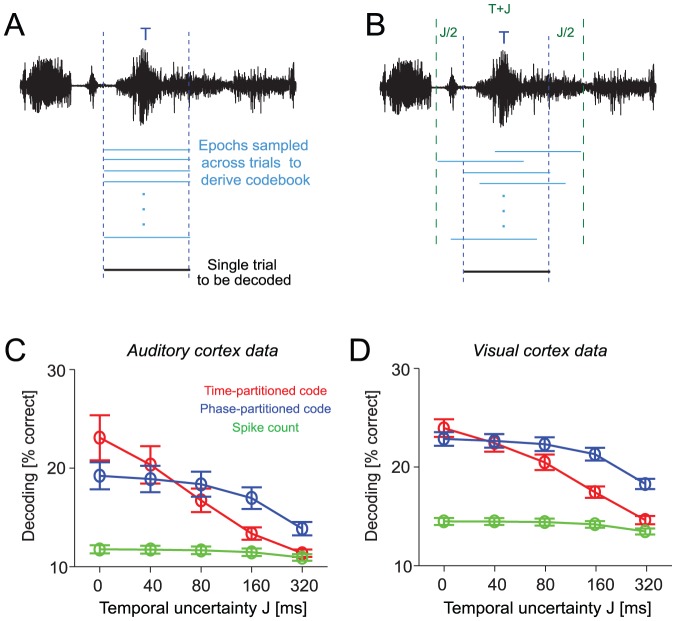
Response partitioning in face of temporal uncertainty. A) Schematic of single trial epoch selection for the decoding process assuming a perfect temporal alignment across trials. When composing the codebook for decoding, the epochs for individual trials are all sampled at the same position relative to the stimulus presentation (blue). Hence the reference epoch in the codebook (blue) and the to-be-decoded single trial (black) are in perfect temporal alignment. B) Schematic for a decoding process introducing a temporal uncertainty (jitter) between trials when composing the codebook. The data epochs for individual trials were shifted by a lag value that was randomly sampled for each trial and which was uniformly distributed between −J/2 and +J/2, where J corresponds to the (maximal possible) temporal uncertainty. C) Decoding performance as a function of temporal uncertainty J for auditory cortical data (n = 40 units, T = 160 ms, N = 8, 2–6 Hz LFP). D) Decoding performance as a function of temporal uncertainty J for visual cortical data (n = 37 units, T = 160 ms, N = 8, 2–6 Hz LFP).

The above analysis has one potential limitation. While within the coding window T spikes are grouped using either partitioning scheme the analysis assumes that the time windows T themselves are perfectly aligned relative to each other across trials. Thereby we assume that the decoder can compare a single trial response with the across-trial distribution of responses at exactly the same position in the stimulus time course (Fig. 5A); i.e. the decoder hence relies on a ‘codebook’ (the set of all single-trial responses) that is sampled at a fixed position relative to the stimulus. This may not be a realistic scenario for actual sensory cortical networks [40], [41]. A downstream decoder might not know the post-stimulus time (neither at the millisecond nor the tens of milliseconds scale) at which a response was emitted and hence may not have access to the ideal codebook used above.

We tested the robustness of the different codes to temporal uncertainty about the precise response alignment in the decoding process. Specifically, we simulated temporal uncertainty by incorporating a jitter Δt (randomly drawn in each trial from a uniform distribution between −J/2 and J/2, J being the degree of maximal uncertainty) in the alignment of responses across trials when deriving the codebook (Fig. 5B). We then evaluated the decoding performance for a range of values of the maximal time shift. This directly probes the robustness of each code to errors made by downstream decoders due to temporal uncertainty in the alignment of single trial responses and sensory events, and therefore provides a crucial test for the computational viability of neural codes under these more stringent and probably more realistic conditions.

We found that the phase-partitioned code was more robust to temporal uncertainty than the time-partitioned code, both in the visual and auditory datasets (Figs. 5C,5D, N = 8bins, T = 160 ms). Decoding performance in each code decreased with increasing uncertainty J, but this decrease was largest for the time-partitioned code. For temporal uncertainties of J = 80 ms or larger (i.e. half the coding window T) the phase-partitioned code provided significantly higher stimulus discrimination than the time-partitioned code (J = 80 ms, auditory data: time-partitioned 16.7±1.1%, phase-partitioned 18.3±1.2%; t-test p<0.01; visual data: time-partitioned 20.5±0.8%, phase-partitioned 22.4±0.7%; p<10^−4^) and this difference was further enhanced for larger temporal uncertainties (Fig. 5C,5D). This demonstrates that oscillatory phase provides a reference to partition temporal response patterns in a manner that is robust to temporal uncertainty in the decoding process and hence excels under conditions which are likely to be present in real cortical networks.

### Stimulus encoding in face of sensory noise

**Figure 6:**
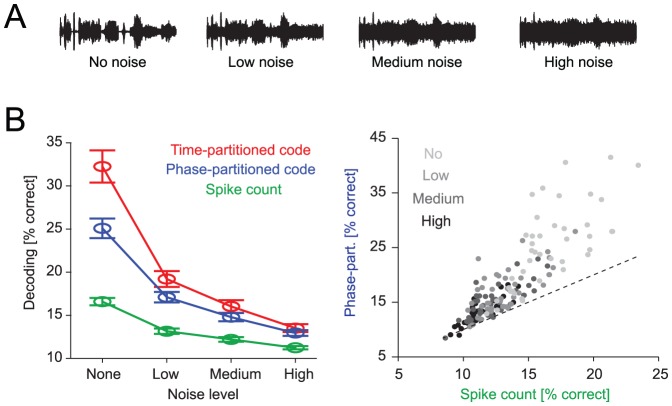
Auditory coding in the presence of background noise. A) Schematic of sound waveforms showing the target sound without background noise and with background noises of three levels. B) Left: Decoding performance across neurons (n = 43 units, mean and s.e.m.) for the three different codes as a function of noise level. Right: Decoding performance with the spike count and the phase-partitioned code for individual neurons (N = 8 bins, T = 160 ms, 2–6 Hz LFP).

Any neural code that is to operate under realistic conditions must not only provide information about clearly perceivable stimuli but must also be robust to noise in the sensory environment that often compromises stimuli of interest. We therefore performed additional analyses to directly quantify the impact of sensory noise on the different codes. Specifically, we analyzed auditory cortical data recorded using a stimulus set where a naturalistic target sound-sequence was systematically degraded by adding background noise [20]. The background noise consisted of a cacophony of natural sounds and a different noise mixture was added on each trial (Fig. 6A). The same target sound was presented either without noise or with noise of three different levels (labeled ‘low’, ‘medium’, and ‘high’; +6, 0 and −6 dB r.m.s. intensity relative to the target sound).

Decoding performance for all codes was reduced by the presence of background noise, and the reduction in performance was greater for higher levels of noise (Fig. 6B, n = 43 units; N = 8bins, T = 160 ms). In all conditions the time-partitioned code provided the highest decoding performance. However, the differences between codes reduced with increasing noise level. In the absence of background noise the average decoding performance differed significantly between all codes (pair-wise t-tests, at least p<0.01). For the highest noise level, however, the phase-partitioned and time-partitioned codes provided comparable levels of decoding (13.5±0.5%, 12.9±0.3%, p>0.05), and significantly more than the spike count (11.2±0.2%, both p<0.001). This shows that the phase-partitioned code provides robust and high levels of stimulus discrimination performance in face of external sensory noise, which is ubiquitous in every-day sensory scenarios.

### Robustness to choice of decoding algorithm

We confirmed that the above results were insensitive to the particular choice of decoding algorithm used for single-trial evaluation of the different codes. To this end we repeated the analysis with a range of single-trial classification algorithms (c.f. Materials and Methods). Note that this does not concern the definition of neural codes, but concerns only the specific choice of classification algorithm used to quantify the degree of single-trial stimulus discrimination afforded by each neural code. Supplemental Fig. S2C shows the results for the auditory cortical dataset. While there was some variation in performance level across classifiers for each given code, the relationships between the three neural codes were preserved for each decoding algorithm, and there was no combination of classifiers for which the performance in the spike count was greater than in the phase-partitioned code. In addition, the results regarding robustness of the different codes in the face of temporal uncertainty or sensory noise were also unaffected by the specific choice use of classifier. Overall we found that for the given size of typical neural datasets (here 30–50 trials per stimulus), simpler algorithms with higher bias but lower variance (e.g. nearest mean or Poisson naïve Bayes) performed slightly better than algorithms with more parameters, and hence lower bias but higher variance (e.g. k-nearest neighbors or multinomial naïve Bayes). This suggests that for applications such as the evaluation of neural codes on experimental data simple decoding algorithms such as template matching are among the best choices and have the additional advantage of being extremely efficient computationally.

## Discussion

Temporal patterns of neural activity on the scale of a few to tens of milliseconds can encode substantial amounts of information not available in spike counts over longer epochs [14], [15], [23], [42], [43], [44], [45]. While behaviorally relevant information and its associated neural responses may extend over time scales of hundreds of milliseconds, the crucial units of information in neural responses often consist of finely timed spike patterns. How the brain partitions responses into such units of information and encodes their temporal order is of great interest for constructing detailed models of sensory encoding and the design of prosthetic devices. The need to partition spike trains becomes especially prominent when considering that the brain has to rely on an internal reference frame to align and decode responses relative to potential sensory inputs [6], [24], [26], [46], [47]. The fact that rapidly varying spike patterns often coexist with informative network oscillations on slower time scales has led to the suggestion that these oscillations may act as a clock to partition spike trains [8], [10], [19], [35]. The work presented here provides a quantitative and comprehensive evaluation of this partitioning scheme in the context of natural visual and auditory stimuli and in a wide range of realistic constraints, such as the presence of sensory noise or in face of temporal uncertainty in the decoding process.

Our analysis revealed that dividing theta cycles into phase quadrants (corresponding to a time scale of a few tens of milliseconds) is sufficient to achieve high levels of stimulus discrimination from phase-partitioned responses. This time scale is in good agreement with previous reports of the precision of neurons in auditory and visual cortices during the presentation of natural stimuli [14], [15], [48], [49] and falls within the range of neural membrane time constants [50], [51], [52]. This makes it realistic that individual phase quadrants could indeed serve as integration epochs for downstream decoders, with individual phase quadrants constituting distinct computational units [13], [53], [54].

### Roles of slow rhythms for information coding

Our work builds on the previous finding that slow cortical rhythms entrain to the dynamics of sensory stimuli [10], [34]. More precisely, previous work studying sound driven low frequency oscillations in auditory cortex using MEG or EEG [33], [38], [39] or intracranial recordings [20], [34] has shown that low frequency oscillations are entrained and phase-locked to repetitive or complex time-varying sounds. As a result of this entrainment the phase of these oscillations becomes reliably time-locked to the stimulus during epochs of dynamic changes in the sensory environment [38], and the phase angle of the oscillation becomes an effective network-intrinsic copy of the stimulus-locked time axis. Previous studies showed that this entrainment is particularly strong in delta and theta bands (i.e. below about 8 Hz) [33], [38], [39]. Our results of highly correlated stimulus decoding performance in time- and phase-partitioned responses and the correlation between oscillatory phase coherence and decoding using phase-partitioned responses show that this alignment of oscillations to sensory inputs is the key component of why a phase-partitioned code successfully recovers a high proportion of the stimulus information available in responses partitioned using a highly precise external clock.

Previous work from our group has reported that the instantaneous phase of slow LFP fluctuations carries information largely complementary to that carried by stimulus-locked spike patterns defined on short time scales of few tens of ms [20], [21]. While this previous work already highlighted the potential importance of spike-phase relations for neural coding, there are key differences to the present study. The previous work treated spike-patterns in a strictly stimulus-locked manner, because it quantified spike patterns by measuring inter-spike intervals with millisecond precision and directly relative to the sensory input, i.e. corresponding to the time-partitioned code used here. In contrast, in the present work we consider how the oscillatory phase can be used to partition longer spike trains into short and stimulus-informative patterns without using a millisecond-precise and stimulus locked clock to measure inter-spike intervals or to form equally spaced time bins. The complementary nature of spikes and network oscillations has also been explored in the hippocampus, where the instantaneous phase was used as a complementary signal to enhance the information derived from the combined neural signal about the animal's position in space [18], [19].

How can one reconcile the previous finding that the instantaneous phase of slow network fluctuations carries information complementary to spike rates computed in short time epochs with the suggestion that the phase of slow rhythms can be used as a network-intrinsic temporal reference frame for partitioning spike patterns extending over hundreds of ms? One possible explanation stems from the fact that the phase of slow oscillations is likely to reflect changes in the local excitation-inhibition balance entrained to slow variations in the sensory environment [10], [53], [55]. Modeling studies show that in recurrent and balanced networks the slow oscillatory phase reflects the network entrainment by slow input dynamics whereas spike rates and spike patterns defined on short time scales reflect the instantaneous strength of the network input [56]. In this view, slow network fluctuations reflect patterns of stimulus dynamics over longer time scales while faster variations in spike patterns encode instantaneous values of specific sensory features. This theoretical framework can provide explanations for both our previous and current findings about the role of slow oscillatory phase in sensory coding. On the one hand, it predicts that the entrainment of low network fluctuations to the stimulus time course makes them a good “time axis surrogate” over scales compatible with the cycle of the slow oscillations (as we found here). On the other hand, it also predicts our previous observation that the oscillatory phase at any given time provides information complementary to that carried by the rate or spike patterns defined on short window [20], [21] because the latter may encode the current value of the stimulus whereas the former reports its temporal position within an excitability cycle. Noteworthy recent work in the olfactory system has shown spikes precisely locked to the rhythmic sniff cycle carry information about different odors [57] and that the animals can discriminate activity patterns occurring at different phases of the sniff cycle [58]. Hence, at least for this system, there is evidence that the relative timing of spikes and oscillatory network activity can be directly exploited to guide behavior.

Previous studies also implicated slow rhythms as a mechanism for binding neural ensembles that collectively encode specific sensory attributes at particular instances in time [8], [11], [59], [60]. While this hypothesis differs from the role as an internal temporal reference for response partitioning, it is possible that the same oscillatory signal could serve as a basis for both grouping processes, whether across time or across a population. Multiplexed spatio-temporal sensory representations across populations of neurons are prominent in sensory cortices and emerging evidence highlights the complexity of population-based codes across multiple scales [6], [12], [17], [47], [61]. Future modeling studies could explore the simultaneous role of oscillations in chunking spike trains into informative units and in dynamically binding ensembles for population coding.

### Oscillatory reference frames provide robustness to temporal uncertainty and sensory noise

Sensory coding in natural environments is complicated by several environmental factors, one being sensory noise that can occlude or corrupt stimuli of interest [62]. We analyzed a dataset designed for testing the performance of neural codes in the presence of sensory noise in the auditory domain and found high noise-robustness in the phase-partitioned code. This robustness is likely a direct result of the prominent entrainment of auditory cortical low frequency oscillations by a wide range of complex sounds [38], [55], [63], suggesting that the prominent entrainment of slow oscillatory activity to the dynamic natural environment might be critical in establishing robust mechanisms of sensory coding.

Another factor that can reduce the ability of neural systems to discriminate sensory stimuli is uncertainty about the occurrence and timing of sensory stimuli. It may be possible for the brain to infer the stimulus timing under specific conditions, such as following well-defined and isolated stimulus onsets [24], [25], [40]. We have previously shown that a subset of neurons in auditory cortex provide a powerful network-intrinsic latency signal that indicates the onset of an unexpected (sudden) stimulus with high precision and fidelity [25]. This signal can be used to construct, in a period of few hundreds of ms following stimulus onset, informative spike patterns without relying on explicit and external knowledge about stimulus timing [25]. However, it remains unclear whether such a latency signal can be used as a timing reference during prolonged periods of sensory stimulation. In fact, recent work suggests that the spike timing of sensory cortical neurons is very precise immediately after stimulus onset, but their precision degrades after some few tens of ms from stimulus onset [64], [65]. In addition, while deriving an intrinsically defined reference point for stimulus onset, our previous work still relied on the millisecond-precise knowledge about the timing of subsequent spikes relative to this reference point, *i.e.* we exploited equally-spaced time bins that were derived using a ‘perfect’ clock [25]. However, it is unlikely that the brain can keep a millisecond-precise representation of time intervals for prolonged periods of continuously evolving naturalistic sensory stimuli. As we show here, slow oscillations may provide a temporal reference to overcome this problem on longer scales. To further understand the putative reference frames provided by oscillatory network activity and population spiking responses, modeling studies on coordinated excitability changes in large-scale networks as well as physiological recordings will be required.

Knowledge about the relative timing of sensory events and neural responses is not only required for partitioning longer response into informative units, but also when explicitly decoding temporally extended response patterns with regard to potential sensory inputs. A downstream decoder that lacks knowledge about the exact post-stimulus time at which a given response was emitted may not be able to access the perfect codebook used in conventional analysis, but rather may rely on a temporally ‘blurred’ version of it. We investigated the robustness of neural codes to such temporal uncertainty by systematically incorporating a temporal jitter at the single-trial level. We found that slow oscillations provide a reference frame that provides significantly greater robustness to temporal uncertainty than stimulus-locked partitioning. This robustness likely results from the locking of individual spikes to cortical oscillatory rhythms [47], [63], which is independent of the alignment of either signal relative to the stimulus.

All in all, our quantitative comparisons highlight key computational advantages provided by partitioning spike trains using oscillatory reference frames that prevail especially during those conditions were sensory evidence is impoverished due to noise and uncertainty.

### Implications for computational models of clock-free transmission and spike timing information

Most existing models exploring the encoding of information in the relative timing of spikes and oscillatory activity were developed to explain the prominent theta phase precession observed in hippocampal data [27], [66], [67]. However, Nadasdy [30], [68] recently proposed a unifying model that uses phase-coding for the transmission and binding of information across the thalamo-cortical and limbic systems. This model highlights the necessity that similar frequencies are used as internal clocks within and across structures in order for phase-based coding to operate efficiently within the brain [30], [68]. While hippocampal and entorhinal neurons lock mainly to theta rhythms, classical studies on sensory cortices have mostly emphasized the locking of sensory neurons to gamma-band oscillations [5], [11], [30]. Our finding that theta band oscillations in primary visual and auditory cortices can be used as reference frame to effectively partition spike trains suggests that slow oscillations may be sufficiently wide-spread to meet the consistency demands for phase-based exchange of information across cortical and sub-cortical structures.

Low frequency oscillations serving as temporal reference frames may also form a crucial component for plasticity in down-stream synapses. Recent work has shown that embedding firing patterns within an oscillatory cycle facilitates downstream learning and decoding with spike timing-dependent plasticity (STDP). Simulation studies show that model neurons equipped with STDP robustly detect a pattern of input currents encoded in the phase of a subset of its afferents, even when these patterns are presented at unpredictable intervals [69]. While in principle STPD rules can be adapted to learn sequences of precise inter-spike-intervals [70], learning patterns referenced to the phase of oscillatory activity facilitates learning even when only a fraction of afferents are organized according to the phase [69]. The present results together with such modeling studies underline the flexibility and power of phase-based reference frames, paving the way for a general framework to quantify the performance of neural codes through entire encoding-decoding and learning chains.

## Materials and Methods

### Ethics statement

The data analyzed here was obtained as part of previous studies [20], [71]. Recordings were obtained from the auditory and visual cortices of adult rhesus monkeys (Macaca mulatta) using procedures described below. All procedures were approved by local authorities (Regierungspräsidium Tübingen) and were in full compliance with the guidelines of the European Community (EUVD 86/609/EEC) and were in concordance with the recommendations of the Weatherall report on the use of non-human primates in research [72]. Prior to the experiments a form-fitting headpost and recording chamber were implanted under aseptic surgical conditions and general balanced anesthesia [73]. As a prophylactic measure antibiotics (enrofloxacin, Baytril) and analgesics (flunixin, Finadyne vet.) were administered for 3–5 d post-operatively. The animals were socially (group-) housed in an enriched environment, under daily veterinary supervision and their weight as well as food and water intake were monitored.

### Recording procedures, data extraction and sensory stimuli – Auditory cortex data

As described in more detail previously [14], [20], neural activity was recorded from caudal auditory cortex of three alert animals using multiple microelectrodes (1–6 MOhm impedance), high-pass filtered (4 Hz, digital two pole Butterworth filter), amplified (Alpha Omega system) and digitized at 20.83 kHz. Recordings were performed in a dark and anechoic booth while the animals were passively listening to the acoustic stimuli. Recording sites were assigned to auditory fields (primary field A1 and caudal belt fields CM, CL) based on stereotaxic coordinates, frequency maps constructed for each animal and the responsiveness for tone vs. band-passed stimuli [74]. Spike-sorted activity was extracted using commercial spike-sorting software (Plexon Offline Sorter) after high-pass filtering the raw signal at 500 Hz (3rd order Butterworth filter). For the present study only units with high signal to noise (SNR>8) and less than 2% of spikes with inter-spike intervals shorter than 2 ms were included. Field potentials were extracted from the broad-band signal after sub-sampling the original recordings at 1 ms resolution. Different frequency ranges of the LFP were extracted as described below.

Acoustic stimuli (average 65 dB SPL) were delivered from two calibrated free field speakers (JBL Professional) at 70 cm distance. For the present study we analyzed auditory cortical data from two experiments, conducted as part of a previous study [20]. The first set of neurons was recorded during the presentation of a continuous 52 s sequence of natural sounds. This stimulus sequence was created by concatenating 21 snippets, each 1–4 s long, of various naturalistic sounds, without periods of silence in between (animal vocalizations, environmental sounds, conspecific vocalizations and short samples of human speech). In the second experiment a 15 s section of the long natural sound was presented either in its original form or mixed with background noise of three different levels. The background noise was obtained by randomly averaging many snippets of natural sounds, resulting in a cacophony-like noise stimulus with a similar power spectrum as the long natural sound, but devoid of clearly discernible sound objects. To quantify the relative contribution of this background noise to the stimulus on individual trials, we used the relative intensity (root mean square intensity over the full 15 s) of the target stimulus relative to that of the background expressed in units of dB [75], [76]. Specifically, the original sound was mixed with background noise of three relative levels: 6 dB softer than the original sound (‘low noise’), the same level as the original sound (‘medium noise’) and 6 dB louder (‘high noise’). Importantly, to resemble true noise a different background noise was randomly generated for each trial. For both datasets each stimulus was repeated many times (on average about 50 repeats of the same stimulus, range 39 to 70 repeats).

### Recording procedures, data extraction and sensory stimuli – Visual cortex data

As described in more detail previously [71], neural activity was recorded from the opercular region of primary visual cortex of two animals while the animals were anaesthetized (remifentanyl, 1 µg/kg/min), muscle-relaxed (mivacurium, 5 mg/kg/h) and ventilated (end-tidal CO2 33 mmHg, oxygen saturation >95%). Body temperature was kept constant and lactated Ringer's solution supplied (10 ml/kg/h). Vital signs (SpO2, ECG, blood pressure, endtidal CO2) were continuously monitored. Signals were recorded using microelectrodes (FHC Inc., Bowdoinham, Maine, 300–800 k Ohms), high-pass filtered (1 Hz, digital two pole Butterworth filter), amplified using an Alpha Omega amplifier system (Alpha Omega Engineering) and digitized at 20.83 kHz. Spike-sorted activity was extracted using the online available Matlab-based spike-sorting software Wave_Clus (http://www.vis.caltech.edu/~rodri/Wave_clus/Wave_clus_home.htm) after high-pass filtering the raw signal at 500 Hz (3rd order Butterworth filter). Field potentials were extracted from the broad-band signal after sub-sampling the original recordings at 1 ms resolution. Different frequency ranges of the LFP were extracted as described below.

Binocular visual stimuli were presented at a resolution of 640×480 pixels (field of view: 30×23 degrees, 24 bit true color, 60 Hz refresh) using a fiber optic system (Avotec, Silent Vision, Florida). Stimuli consisted of ‘naturalistic’ complex and commercially available movies (30 Hz frame rate; (Star Wars Episode 4 and The Last Samurai), from which 240 s long sequences were presented and repeated 30–40 times.

### Extraction of frequency bands from field potentials

For the main analysis we extracted individual frequency bands (4 Hz width) from the broadband signal using 3^rd^ order Butterworth filters. The phase angle of the narrow-band signal was subsequently determined using the Hilbert transform. We systematically tested frequency bands with center frequencies from 4 to 32 Hz. In additional control analysis we made sure that our results do not depend on this specific choice of filter. In particular, we compared the performance of the phase-partitioned code using four different filters to derive the theta range: 1) 3rd order Butterworth filter between 2–6 Hz; 2) the same filter to derive a broader frequency band of 2–10 Hz; 3) Kaiser window FIR filter between 2–6 Hz (1 Hz transition bandwidth, passband ripple of 0.01 dB and stopband attenuation of 60 dB; 3) Kaiser window FIR filter 2–8 Hz (2 Hz transition bandwidth, passband ripple of 0.01 dB and stopband attenuation of 30 dB); 5) Morlet wavelet filtering (4 Hz center frequency, standard deviation σf of 0.6/4 Hz). The stimulus decoding performance of the phase-partitioned code changed only minimally with the filter (Supplemental Fig. S2A).

### Definition of neural codes

We quantified the level of stimulus discrimination afforded by three hypothetical neural codes, each derived from the same neural response in the same time window. We defined neural codes in time windows of length T, whereby T was chosen to roughly match the duration of one cycle of the considered oscillation, i.e. T = 160 ms for the 2–6 Hz frequency band (Fig. 1). Given the spiking activity and LFP within this time window, we defined the following three codes. 1) The *time-binned firing-rate*, which was defined by splitting the response window T into N precisely aligned, equally spaced time bins and counting the number of spikes occurring in each bin. Formally, this code can be described as 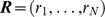 with r_i_ being the number of spikes within the *i*-th time bin, with the bins being defined as the time intervals ([0,T/N],…[(N−1)*T/N,T]). 2) The *distribution of phase of firing*, defined by splitting the phase of the slow reference rhythm into N equally spaced phase bins and allocating each spike to the bin corresponding to the instantaneous phase value at the time of the spike. Formally, this can be described as 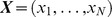 with x_i_ being the number of spikes within the *i*-th phase bin, with the bins being defined as the phase intervals ([0,2*pi/N],…[(N−1)*2*pi/N,2*pi]). 3) We defined the *spike count* as the total number of spikes per time window T, regardless of their temporal sequence. Practically, we implemented the spike count using two different procedures, which yielded very similar results. One procedure was based on shuffling (independently for each trial and time window) the time bins of the time-partitioned code. This shuffling effectively destroys the temporal response pattern and preserves only the total spike count. Formally, this code can be described as 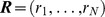 with r_i_ being the number of spikes within a randomly selected (without replacement) time bin. The decoding performance for the spike count using this shuffled procedure was obtained by averaging performance from several repeated computations, to minimize the effect of shuffling (20 times). This shuffling reproduces the information contained in the total spike count, but preserves the dimensionality of the neural code, which is important to ensure the comparability of results obtained in the decoding analysis [14]. In addition, we also implemented the spike count using a 1-dimensional representation, which yielded very similar results as obtained using the shuffling procedure (Supplemental Fig. S2B).

The neural codes defined by the time- and phase-partitioned responses include differences in the spike count. As a consequence, the stimulus discrimination performance afforded by these two codes includes discrimination performance afforded by differences in spike count alone and hence the decoding performance using these two codes is at least as high as from the spike count. The excess decoding performance in each partitioning scheme over that provided by the spike count reflects the information that the respective code carries above and beyond that provided by the spike count [6], [37]. We quantified this excess performance by calculating the difference in decoding performance between each partitioning scheme and the spike count (e.g. Fig. 3B, 4C). We also directly compared the relative excess performance in both partitioning schemes for each neuron by expressing the excess performance in the phase-partitioned code relative to that in the time-partitioned code as percentage.

To more directly quantify the overlap of the decoding performance permitted by time- and phase-partitioned codes we also performed an analysis on their decoding redundancy. We created a dual-code based on the joint response of both time- and phase-partitioned response vectors. Formally this code can be represented as 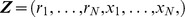, with r_i_ being the number of spikes within the *i*-th time bin and x_i_ being the number of spikes within the *i*-th phase bin. The decoding performance of this dual-code should exceed the performance of the individual codes by an amount proportional to the degree of separate stimulus discriminability afforded uniquely by each code. We expressed the relative performance of the dual code to the better of the two individual codes as a percentage.

For each dataset we quantified the performance of the different codes for a wide range of parameters for the time window T (80 to 480 ms), the number of bins N (2 to 16) and the frequency band of the local field potential from which the oscillatory phase was extracted (center frequencies from 4 to 32 Hz). To quantify the impact of frequency band on performance of the phase-partitioned code, we computed the ratio of decoding performance between the phase-partitioned code and the spike count after subtracting the chance performance. If not stated otherwise, results refer to a ‘standard’ choice of T = 160 ms, N = 8 and the 2–6 Hz band.

### Single trial decoding and stimulus information

We quantified the stimulus discrimination afforded by each code using a single-trial decoding procedure, applied to the responses in ten epochs of duration T. These epochs were randomly sampled (non-overlapping) from within the entire stimulation period (Fig. 2A) and the decoding procedure was averaged over 100 independent sets of epochs.

For decoding we used linear template matching in conjunction with a leave-one-out cross-validation procedure [32], [77]. For each individual trial from a given stimulus (say S1) this proceeded as follows: 1) The average responses to all other 9 stimuli were computed by averaging the responses of all repeats of the respective stimuli. 2) For the current stimulus (S1) the mean response was computed by averaging across all trials, excluding the current ‘test’ trial. These average responses to each stimulus then represented the ‘codebook’. 3) The Euclidean distance was computed between the response vector (representing spike counts in time or phase bins) on the test trial and the average responses in the codebook. The test trial was decoded as that stimulus yielding the minimal distance to the test response.

Formally, let 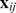 denote the vector representing one of the neural codes for the presentation of stimulus *i* on trial *j*, and let 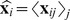 denote the mean response to stimulus *i* (computed excluding the current leave-one-out test trial). The test trial 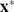 is decoded as the stimulus index *i* whose mean distance to the test trial is smallest:
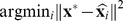
(1)This nearest mean or template matching procedure can be shown to be the Bayes optimal decoder in the case where the stimulus conditional response ensembles are independent multivariate normal distributions (constant diagonal covariance). This procedure was repeated for each trial of each of the 10 stimuli and the total percentage of correctly decoded trials and the confusion matrix were determined. To quantify the performance of the decoder we used the percent of correctly identified trials and averaged this measure over 100 sets of independently sampled epochs.

To validate that our results do not depend on this specific choice of single trial decoding algorithm we repeated the entire analysis using a wider range of classification algorithms [78]. Specifically, we implemented a range of classifiers that permit an efficient implementation of the above leave-one-out cross validation procedure. These classifiers were: (1) the above described nearest mean template matching procedure, which corresponds to an optimal linear discriminant classifier in the case where the stimulus conditional response distributions are multivariate normal, independent, and with equal variances. These assumptions inherent to the nearest mean classifiers can be progressively relaxed, leading to classifiers estimating the covariance matrix pooled across stimuli (2: linear classifier), estimating the full covariance matrix for each stimulus (3: quadratic classifier). For these classifiers, in order to avoid numerical problems due ill-conditioned matrices we added a small random jitter (normally distributed with standard deviation 0.001) to the discrete count responses independently for each trial and bin. We verified that this procedure did not affect results for repetitions without numerical problems, and that adding the jitter produced similar results to the more computationally intensive use of the matrix pseudo-inverse. In addition we implemented Naïve Bayes decoders, which assume that response variables (e.g. counts in each time or phase bin) are independent and calculate the most likely stimulus using Bayes theorem. We implemented (4) Poisson naïve Bayes, which assumes the counts are Poisson distributed, and (5) multinomial naïve Bayes, which samples the full discrete stimulus-conditional probability distributions of counts for each response bin. Finally (6) we also implemented a k-nearest neighbor classifier with Euclidean distances.

### Decoding with temporal uncertainty

When computing stimulus information in face of temporal uncertainty about the precise alignment of individual time windows T to the sensory stimulus, we added a jitter to the alignment of time windows across trials when calculating the codebook (Fig. 5A,5B). Specifically, we randomly shifted the window extracted for each trial by a random lag into the future or past that was randomly sampled for each trial and which was uniformly distributed between −J/2 and +J/2, where J corresponds to the (maximal possible) temporal uncertainty. These time-shifted single trial responses were then averaged to obtain the codebook (steps 1 and 2 in the decoding process). Again the entire process was repeated 100 times using different selection of stimulus epochs.

### Oscillatory phase coherence

We calculated the trial-by-trial coherence of the oscillatory phase using the inter-trial phase coherence index. This index is a measure of phase concentration across trials and is defined as
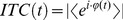
(2)where <.> denotes the trial average, φ(t) the phase at time t and |.| the absolute value of the complex number. To compute the correlation of oscillatory phase coherence and decoding performance (Fig. 3D) we averaged phase coherence across time points within the decoding window T, resulting in a single phase coherence value for each stimulus epoch. This value was then correlated with the decoding performance for each of the considered codes across stimulus epochs.

## Supporting Information

Figure S1 Dependency of decoding performance on window length T and the number of bins N. ‘Ribbons’ display the population average performance for the auditory dataset. Fig. 3E of the main manuscript shows two individual sections (at fixed N = 8 and at fixed T = 160 ms).
(TIF)

Figure S2 A) The decoding performance of the phase-partitioned code is independent of the specific parameters and filters used to derive the LFP band. The bars display the decoding performance for the auditory dataset (c.f. Fig. 3A, N = 8 bins and T = 160 ms window, 2–6 Hz band) for four different filter parameters: 1) 3rd order Butterworth filters between 2–6 Hz; 2) 3rd order Butterworth filters between 2–10 Hz; 3) Kaiser filters between 2–6 Hz (1 Hz transition bandwidth, passband ripple of 0.01 dB and stopband attenuation of 60 dB); 4) Kaiser filters between 2–8 Hz (2 Hz transition bandwidth, passband ripple of 0.01 dB and stopband attenuation of 30 dB); 5) Morlet wavelet filtering (4 Hz center frequency, standard deviation of 0.6/4 Hz). B) The decoding performance of spike count code is independent on whether the code is implemented using a N-dimensional response vector whose elements are shuffled (randomly across trials and stimuli) or whether the code is implemented using a 1-dimensional number. The bars display the decoding performance for the auditory dataset (c.f. Fig. 3A, N = 8 bins and T = 160 ms window). C) Robustness of results to choice of single-trial decoding algorithm. We repeated the analysis of the auditory cortex data (c.f. Fig. 3A) using 6 different algorithms for the single trial decoding procedure. The figure displays the average decoding performance for each algorithm (n = 40) for each of the three codes. The relative differences between different neural codes, and hence our main result, was preserved across all tested classification algorithms. Dashed lines indicate that no combination of classifiers and neural codes would change our main findings (e.g. phase-partitioning to be superior to spike counts).
(TIF)
