# Supplementary figures and images for: Analysis of Slow (Theta) Oscillations as a Potential Temporal Reference Frame for Information Coding in Sensory Cortices
Source: PLoS Comput Biol. 2012 Oct 11;8(10):e1002717. doi: 10.1371/journal.pcbi.1002717 (PMC3469413; doi:10.1371/journal.pcbi.1002717)

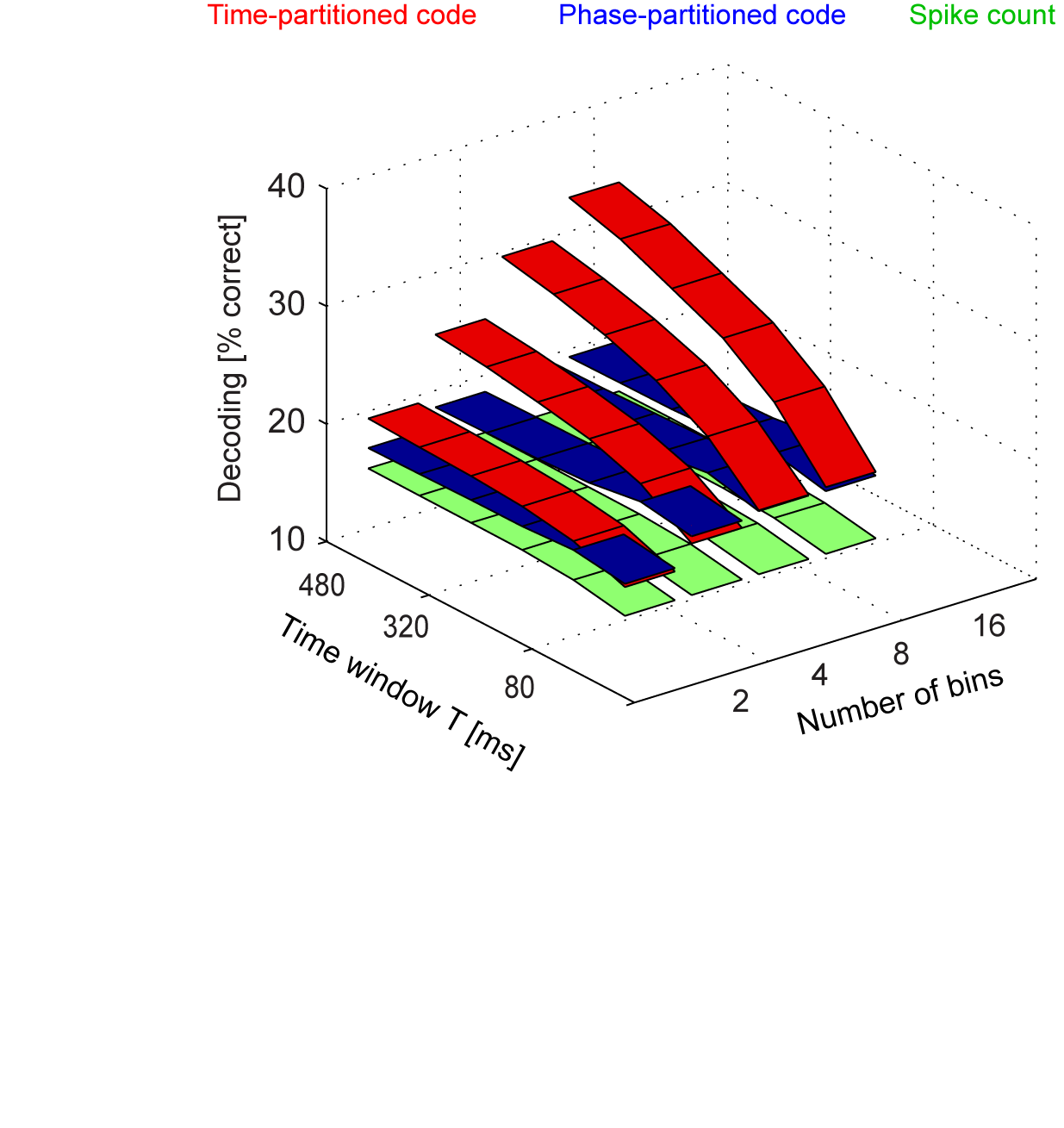

Supplement: Figure S1 — Dependency of decoding performance on window length T and the number of bins N. ‘Ribbons’ display the population average performance for the auditory dataset. Fig. 3E of the main manuscript shows two individual sections (at fixed N = 8 and at fixed T = 160 ms). (TIF) [file pcbi.1002717.s001.tif]

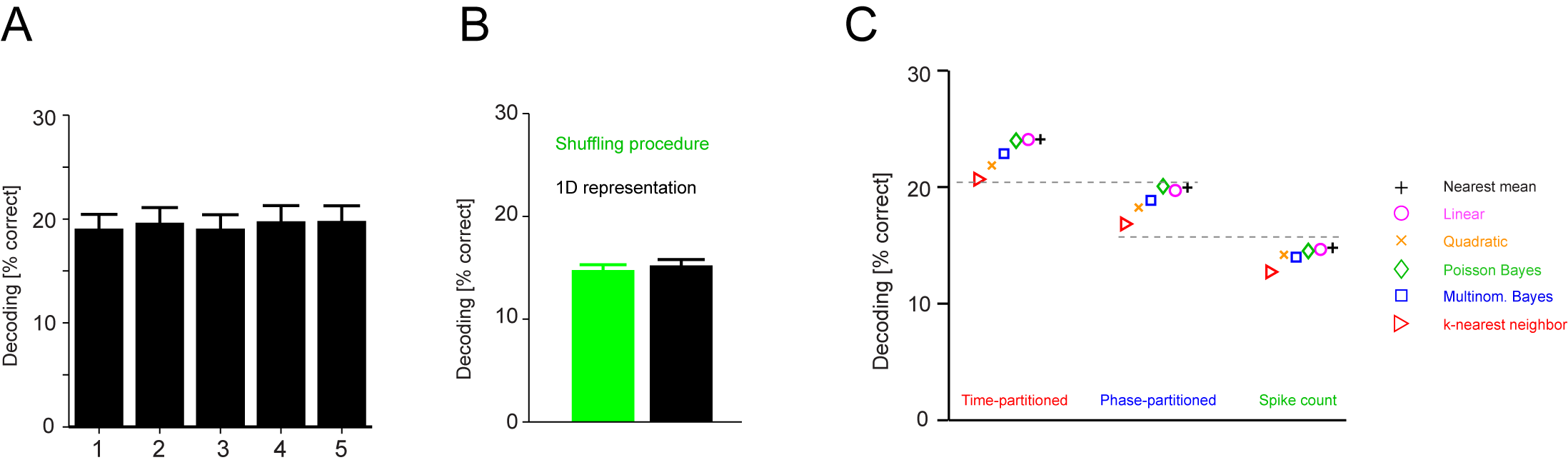

Supplement: Figure S2 — A) The decoding performance of the phase-partitioned code is independent of the specific parameters and filters used to derive the LFP band. The bars display the decoding performance for the auditory dataset (c.f. Fig. 3A, N = 8 bins and T = 160 ms window, 2–6 Hz band) for four different filter parameters: 1) 3rd order Butterworth filters between 2–6 Hz; 2) 3rd order Butterworth filters between 2–10 Hz; 3) Kaiser filters between 2–6 Hz (1 Hz transition bandwidth, passband ripple of 0.01 dB and stopband attenuation of 60 dB); 4) Kaiser filters between 2–8 Hz (2 Hz transition bandwidth, passband ripple of 0.01 dB and stopband attenuation of 30 dB); 5) Morlet wavelet filtering (4 Hz center frequency, standard deviation of 0.6/4 Hz). B) The decoding performance of spike count code is independent on whether the code is implemented using a N-dimensional response vector whose elements are shuffled (randomly across trials and stimuli) or whether the code is implemented using a 1-dimensional number. The bars display the decoding performance for the auditory dataset (c.f. Fig. 3A, N = 8 bins and T = 160 ms window). C) Robustness of results to choice of single-trial decoding algorithm. We repeated the analysis of the auditory cortex data (c.f. Fig. 3A) using 6 different algorithms for the single trial decoding procedure. The figure displays the average decoding performance for each algorithm (n = 40) for each of the three codes. The relative differences between different neural codes, and hence our main result, was preserved across all tested classification algorithms. Dashed lines indicate that no combination of classifiers and neural codes would change our main findings (e.g. phase-partitioning to be superior to spike counts). (TIF) [file pcbi.1002717.s002.tif]
